# Effects of Viral Infections Like COVID-19 on Head and Neck Cancers: The Role of Neutrophil-Lymphocyte Counts and Ratios

**DOI:** 10.7759/cureus.61733

**Published:** 2024-06-05

**Authors:** Sunayana R Sarkar, Hitesh R Singhavi, Abhishek Das, Ipsita Dhal, Shreya Shukla, Sambit S Nanda, Aseem Mishra, Komal Lamba, Anamika Mishra

**Affiliations:** 1 Head and Neck Surgery, Homi Bhabha Cancer Hospital and Mahamana Pandit Madan Mohan Malaviya Cancer Centre, Varanasi, IND; 2 Head and Neck Surgery, Fortis Hospital, Mulund, Mumbai, Mumbai, IND; 3 Oncopathology, Homi Bhabha Cancer Hospital and Mahamana Pandit Madan Mohan Malaviya Cancer Centre, Varanasi, IND; 4 Radiology, Homi Bhabha Cancer Hospital and Mahamana Pandit Madan Mohan Malaviya Cancer Centre, Varanasi, IND; 5 Radiation Oncology, Homi Bhabha Cancer Hospital and Mahamana Pandit Madan Mohan Malaviya Cancer Centre, Varanasi, IND; 6 Anesthesiology, Homi Bhabha Cancer Hospital and Mahamana Pandit Madan Mohan Malaviya Cancer Centre, Varanasi, IND

**Keywords:** cancer and nlr, oral cancers, covid-19 nlr, head and neck squamous cell cancer, neutrophil to lymphocyte ratio (nlr)

## Abstract

Background: Over the last three years, the coronavirus disease 2019 (COVID-19), caused by the severe acute respiratory syndrome coronavirus 2 (SARS-CoV-2), has had a global impact. COVID-19 has led to diagnostic and treatment delays in head and neck squamous cell cancers (HNSCCs). Both cancer and COVID-19 trigger systemic inflammatory responses that can result in cytokine storms, creating a favorable tumor microenvironment that supports tumor growth. Various studies have shown a positive association between increasing neutrophil-to-lymphocyte ratio (NLR) and disease severity in COVID-19. Studies have also shown that high NLR is associated with poor survival outcomes in cancer patients. Our aim is to investigate whether an increased NLR is linked to rapid tumor progression in patients with HNSCC who have also been affected by infections like COVID-19 in the pre-operative period.

Methods: This was a retrospective analysis of patients of HNSCC who were scheduled for surgery and had contracted COVID-19 in their pre-operative period between April 2021 and May 2021. The study analyzed pre- and post-COVID NLR in relation to disease progression in HNSCC. Statistical analysis was presented as an interquartile range and numbered with the percentage. Statistical Package for the Social Sciences (IBM SPSS Statistics for Windows, IBM Corp., Version 26.0, Armonk, NY) was utilized for the analysis.

Results: We evaluated 200 operable cases of which 38/200 (20%) patients with HNSCC were COVID-19 positive. Out of those COVID-19-positive patients, 27/38 (71%) patients got operated. Around, 11/38 (28.9%) patients were inoperable. And, 14/27 (53.8%) operated patients also had a change in treatment plan. The mean duration from the joint clinic treatment plan to the date of surgery was 25.18 days. Patients who had contracted COVID-19 and had a change in their treatment plan due to disease progression exhibited mean NLR values of 3.84 (pre-COVID) and 11.11 (post-COVID), with respective medians of 3.04 and 10.50. These differences showed a statistically significant p-value of 0.000. In contrast, patients who had no change in treatment plan displayed mean NLR values of 4.51 (pre-COVID) and 9.70 (post-COVID), with respective medians of 3.47 and 3.42, resulting in with a non-significant p-value of 0.082.

Conclusion: This is a one-of-its-kind study that has evaluated the role of elevated NLR in patients with a COVID-19 virus infection and its relationship with the clinical progression of the disease. The findings suggest that elevated NLR in patients with HNSCC, along with concurrent SARS-CoV2 infection, may contribute to accelerated disease progression with an increase in tumor burden and nodal metastasis.

## Introduction

Severe acute respiratory syndrome coronavirus 2 (SARS-CoV-2) infection has affected the globe, causing the coronavirus disease 2019 (COVID-19) pandemic [1]. As of June 2023, India has surpassed 44 million cases of COVID-19 and reported a death toll of 5.31 lakhs [2]. A surge in COVID-19 cases led to travel restrictions and lockdowns, which negatively affected cancer treatment and elective surgeries [3]. This situation mirrors patterns observed after natural disasters, such as Hurricane Katrina and the Fukushima earthquake, where delayed diagnoses persisted for years [4]. Head and neck cancers can potentially double in volume within one to three months, regardless of their size or site of origin [5]. Regardless of the pandemic, delays in diagnosis and initiation of treatment are known to impact overall survival [6].

The host inflammatory response plays a key role in tumor development and progression in advanced stages of cancer [7]. This chronic inflammatory state has been reported to be associated with increased tumor burden and poor prognosis [8]. COVID-19 infection also triggers inflammatory cytokines and various studies have found severe COVID-19 symptoms to be associated with increased neutrophils and high neutrophil-to-lymphocyte ratio (NLR) [9].

We hypothesized that patients with head and neck cancer who contracted COVID-19 infection may have some alteration in their disease biology. Therefore, we analyzed the patients with head and neck squamous cell cancers (HNSCCs) infected with COVID-19 during the pandemic and correlated their NLR with the clinical disease progression.

## Materials and methods

Study design

This was a retrospective cohort analysis conducted on patients diagnosed with HNSCC and who had contracted COVID-19 in their pre-operative period. All patients were divided into two subgroups. Patients who had a change in treatment plan post-COVID-19 infection in contrast to no change in treatment plan. Their NLR values were evaluated before and after the infection and correlated with the pre- and post-COVID clinical TNM stage. This study was conducted between April 2021 and May 2021 at the beginning of the second wave COVID-19 pandemic, when the incidence of COVID-19 infection was at its peak at our center. Patients who tested negative with reverse transcriptase-polymerase chain reaction (RT-PCR) two weeks after being infected with COVID-19 were re-assessed. Those with the disease amenable to resection either underwent upfront surgery as planned previously in the joint clinic or required a more extensive resection with reconstruction due to disease progression. Those deemed unresectable were treated with palliative intent. This study complied with the ethical guidelines outlined in the Declaration of Helsinki [10]. The study was performed in accordance with the Institutional Ethical Committee of the hospital and the requirement for informed consent was waived by the Ethics Committee. Routine blood examinations consisting of complete blood count, coagulation profile, serum biochemical tests (including renal and liver function), and electrolytes were conducted. All patients underwent chest radiographs or CT scans as a part of the evaluation.

The electronic medical records of the patients were analyzed for demographic, clinical, and laboratory details. The patient’s medical history and comorbidities were recorded. The tumor size and stage were recorded. The staging was done as per the eighth edition of the American Joint Committee on Cancer (AJCC)/Union for International Cancer Control (UICC) staging system.

Inclusion criteria

All patients of HNSCC with available preoperative neutrophil and lymphocyte counts and who had tested positive for COVID-19 after the treatment plan was made were included in this study.

Exclusion criteria

Patients with missing neutrophil and lymphocyte count records, patients with chronic hematological disorder, and patients who contracted COVID-19 infection in the post-operative period were excluded from the study.

All the clinical and laboratory values such as the absolute neutrophil counts (ANC), lymphocyte count, and NLR were recorded. Change or progression in the disease status was recorded as an increase in tumor dimensions, increased levels of neck nodal involvement, or contralateral spread. Any change in the final surgical treatment plan from the previous joint clinic decision was recorded.

Statistical analysis

Baseline characteristics were presented as a median with interquartile range (IQR) for continuous variables and as a number with percentage for categorical variables. Statistical Package for the Social Sciences (IBM SPSS Statistics for Windows, IBM Corp., Version 26.0, Armonk, NY) was utilized for the analysis.

## Results

TNM stage of pre- and post-COVID-19 patients shows the clinical progression of the disease (Table 1). Out of the 200 patients screened, 42 had HNSCC and tested positive for COVID-19. Of these, 40 developed COVID-19 before surgery, while two contracted it after surgery. Two patients were lost to follow-up. Therefore, we included 38 patients in our final analysis.

**Table 1:** Tumor and nodal stage before and after COVID-19 infection in patients with HNSCC HNSCC: head and neck squamous cell cancer

Part A			
Tumor Stage			
Pre-COVID		Post-COVID	
cT1	6	cT1	2
cT2	11	cT2	2
cT3	3	cT3	3
cT4a	18	cT4a	22
cT4b	-	cT4b	9
Nodal Stage			
Pre-COVID		Post-COVID	
cN0	26	cN0	18
cN1-2	10	cN1-2	9
cN3b	2	cN3b	11
Part B			
Reason for in-operability		No. of Patients n=11	
Primary Disease			
Extensive infratemporal fossa involvement		04	
Extensive involvement of base skull		00	
Extensive induration/soft tissue disease till zygoma or hyoid		02	
Nodal Disease			
Clinically fixed nodes		03	
Infiltration of internal/common carotid artery		01	
Extensive infiltration of prevertebral muscles, skull base		01	

Among these three patients had hypertension; one had type II diabetes mellitus (DM); one had both HTN and DM, and one had ischemic heart disease (IHD).

During the waiting period due to COVID-19 infection, 11 out of 38 patients (28.9%) were deemed unresectable because of disease progression and were planned for palliative chemotherapy or radiotherapy options as per the decision made in the joint clinic of head and neck oncology. Twenty-seven out of 38 patients (71.05%) underwent surgery for HNSCC, out of which 14 (51.85%) patients had to undergo a more extensive resection as compared to the plan made prior to contracting COVID-19. Disease progression was seen as an increase in tumor dimensions (57%), increased levels of neck nodal involvement or contralateral spread (42%), or a change in the reconstruction plan that required a larger flap reconstruction. Table 2 compares the histopathological characteristics of patients whose treatment plans changed after contracting COVID-19 to those of patients whose treatment plans stayed the same.

**Table 2:** Comparison of operated patients with a change in the treatment plan ENE: extranodal extension; WPOI: worst pattern of invasion; PNI: perineural invasion; CTRT: chemoradiotherapy

	Unchanged treatment plan N=13	Changed treatment plan N=14
Tumor upstaged	3	6
Nodal disease upstaged	1	6
ENE	1	6
Poor tumor grade	-	1
Aggressive WPOI	3	11
PNI	2	6
Adjuvant CTRT	2	11

Patients who had contracted COVID-19 and had a change in their treatment plan due to disease progression (25/38,65.78%) exhibited mean NLR values of 3.84 (pre-COVID) and 11.11 (post-COVID), with respective medians of 3.04 and 10.50. These differences showed a statistically significant p-value of <0.000. In contrast, patients who had no change in treatment plan (13/38, 34.21%) displayed mean NLR values of 4.51 (pre-COVID) and 9.70 (post-COVID), with respective medians of 3.47 and 3.42, resulting in a with a non-significant p-value of <0.082 (Table 3).

**Table 3:** Pre- and post-NLR ratio of patients whose treatment plan changed after COVID-19 infection as compared to those whose treatment plan remained unchanged NLR: neutrophil-to-lymphocyte ratio

	Pre-NLR ratio		Post-NLR ratio		P-value
	Mean	Median	Mean	Median	
Plan changed (25)	3.8412	3.0400	11.1110	10.5000	0.000
Plan not changed (13)	4.5100	3.47000	9.7046	3.4200	0.082

The mean duration in days from the joint clinic’s treatment plan to the date of surgery was 25 days.

## Discussion

Head and neck cancers constitute a significant portion of global cancer cases, with approximately 57.5% of these cases occurring in Asia, particularly in India, where they account for 30% of all cancer cases [11]. The link between inflammation and carcinogenesis was first proposed by Rudolf Virchow in 1863. Since then, several studies have contributed to the characterization of the microenvironment for a better understanding of tumorigenesis, progression, and treatment. Although cancer had previously been viewed as a heterogeneous disease involving aberrant mutations in tumor cells, it is now evident that tumors are also diverse by nature of their microenvironmental composition, and stromal cell proportions or activation states. Studies on HNSCC pathogenesis have revealed that host inflammatory response has an important role in tumor development and progression [8].

The ongoing COVID-19 pandemic has profoundly impacted healthcare systems worldwide, leading to delays in cancer treatment and a surge in advanced-stage cancer presentations. Although the pathophysiology of COVID-19 is not yet fully understood, it shares commonalities with other viral infections in triggering uncontrolled inflammatory responses, including the release of pro-inflammatory cytokines during a phenomenon referred to as a "cytokine storm." This excessive immune response to SARS-CoV-2 contributes to the severity of COVID-19 [12]. A recent study by Qin et al. showed a higher NLR ratio in patients with severe forms of COVID-19 due to a systemic hyperinflammatory state [13]. Recent studies have also shown a strong association between increased NLR and decreased survival in patients with HNSCC. Therefore, despite being distinct pathologies, both HNSCC and COVID-19 share a crucial aspect of their pathogenesis, in the induction of a "cytokine storm" with an exaggerated hyperinflammatory response.

Neutrophils, traditionally seen as innate immune defenders, have been found to play dual roles, both promoting tumor progression and suppressing cytotoxic T-cell activity, thereby facilitating metastasis [14]. Neutrophilia is commonly accompanied by relative lymphocytopenia, representing a significant decline in the cell-mediated adaptive immune response. The NLR captures the balance between the detrimental effects of neutrophilia and the beneficial effects of lymphocyte-mediated adaptive immunity [15]. Both systemic neutrophilia and lymphopenia are associated with poorer prognosis in cancer patients [16]. Many previous studies have examined the prognostic value of pre-treatment NLR, and strong associations have consistently been demonstrated between high NLR and poor patient outcomes across many cancers [17].

Neutrophils also exhibit heightened responses during viral infections and interact extensively with T-cells, expressing various receptors and adhesion molecules. In COVID-19 patients, increased NLR levels have been documented, and higher values are strongly associated with the disease severity.

In the present study, we found that the majority of HNSCC patients who contracted COVID-19 infection in the pre-operative period exhibited higher NLR values. A total of 65.78% of these patients were seen to have an accelerated disease progression and hence had a change in the treatment plan requiring much more aggressive ablative surgery with larger flap reconstruction or change in the intent to palliation. These patients had significantly elevated NLR compared to the ones who had no change in the treatment plan. This indicates that a higher NLR is strongly associated with disease progression.

Studying the progression of the disease may not be feasible; however, a patient with HNSCC exhibiting elevated NLR or who may have contracted any infection, triggering a systemic hyperinflammatory response that could also elevate NLR, needs to be treated as a priority due to the potential for accelerated disease progression.

Limitations

The sample size of the study is small, as it included the patients who contracted COVID-19 infection and were planned for surgery. It has the limitation of being a retrospective study and a single-center study. The study was analyzed over a short duration of time. Several other inflammatory factors that were not analyzed could also contribute to the disease progression.

## Conclusions

This is a one-of-its-kind study that has evaluated the role of elevated NLR in patients with a COVID-19 virus infection and its relationship with the clinical progression of the disease. The findings suggest that elevated NLR in patients with HNSCC, along with concurrent SARS-CoV2 infection, may contribute to accelerated disease progression with an increase in tumor burden and nodal metastasis. Although COVID-19 was associated with peculiar features, any infection in patients with HNSCC which tends to increase the NLR may portend a poor prognosis and cause an accelerated progression. This needs to be verified in other prospective cohorts of patients. Further follow-up studies are warranted, with special emphasis on inflammatory changes and their role in disease progression, metastasis, and treatment.
